# Loess Plateau storage of Northeastern Tibetan Plateau-derived Yellow River sediment

**DOI:** 10.1038/ncomms9511

**Published:** 2015-10-09

**Authors:** Junsheng Nie, Thomas Stevens, Martin Rittner, Daniel Stockli, Eduardo Garzanti, Mara Limonta, Anna Bird, Sergio Andò, Pieter Vermeesch, Joel Saylor, Huayu Lu, Daniel Breecker, Xiaofei Hu, Shanpin Liu, Alberto Resentini, Giovanni Vezzoli, Wenbin Peng, Andrew Carter, Shunchuan Ji, Baotian Pan

**Affiliations:** 1 Key Laboratory of Western China's Environmental Systems (Ministry of Education), College of Earth and Environmental Sciences, Lanzhou University, Lanzhou 73000, China; 2 Department of Earth Sciences, Uppsala University, Villavägen 16, 75236 Uppsala, Sweden; 3 Department of Earth Sciences, University College London, Gower Street, London WC1E 6BT, UK; 4 Department of Geological sciences, University of Texas, Austin 78712, USA; 5 Department of Earth and Environmental Sciences, University of Milano-Bicocca, Piazza della Scienza 4, 20126 Milano, Italy; 6 Department of Geography, Environment and Earth Sciences, University of Hull, Cottingham Road, Hull HU6 7RX, UK; 7 Department of Earth and Atmospheric sciences, University of Houston, Houston, Texas 77204, USA; 8 School of Oceanographic and Geographic Sciences, Nanjing University, Nanjing 210023, China; 9 Department of Earth and Planetary Sciences, Birkbeck, University of London, Malet Street, London WC1E 7HX, UK

## Abstract

Marine accumulations of terrigenous sediment are widely assumed to accurately record climatic- and tectonic-controlled mountain denudation and play an important role in understanding late Cenozoic mountain uplift and global cooling. Underpinning this is the assumption that the majority of sediment eroded from hinterland orogenic belts is transported to and ultimately stored in marine basins with little lag between erosion and deposition. Here we use a detailed and multi-technique sedimentary provenance dataset from the Yellow River to show that substantial amounts of sediment eroded from Northeast Tibet and carried by the river's upper reach are stored in the Chinese Loess Plateau and the western Mu Us desert. This finding revises our understanding of the origin of the Chinese Loess Plateau and provides a potential solution for mismatches between late Cenozoic terrestrial sedimentation and marine geochemistry records, as well as between global CO_2_ and erosion records.

## Abstract (web summary)

Theories of Cenozoic mountain uplift and associated global cooling assume that eroded mountain sediments are stored in marine basins. Here, based on detailed provenance data, Nie *et al*. show that Northeast Tibetan sediments are in fact stored inland, in the Chinese Loess Plateau and Mu Us desert.

## Graphical abstract

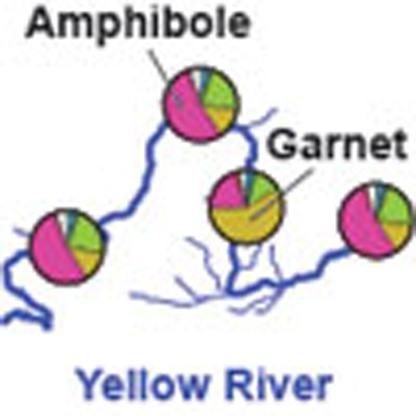

The Yellow River (Fig. 1) currently has the world's highest sediment load^1^, with an annual sediment discharge of 1,080 × 10^6^ ton per year to the ocean. It is therefore a critical link between eroding uplands and terrigenous sediment records of the marine sedimentary basins used extensively to reconstruct orogenic denudation histories^2^,^3^,^4^. The river's sediment sources, transport and dispersal patterns, as well as its formation history^5^,^6^,^7^,^8^, are key for understanding the controversial timing, cause, extent and impact of uplift and denudation of the Northeast (NE) Tibetan Plateau and tectonic–climate linkages^3^,^9^,^10^. However, the origin and drainage history of the river are highly controversial, with estimates of establishment of the current drainage patterns ranging from the Eocene to late Pleistocene^6^,^8^,^11^. Furthermore, the globally important climate and dust archive, the Chinese Loess Plateau loess deposits that lie within the square bend of the Yellow River (Fig. 1), are widely considered to be derived solely from aeolian transport of dust directly from source regions in NE Tibet, western China or northern China and Mongolia^12^,^13^,^14^,^15^,^16^,^17^,^18^,^19^,^20^. The Yellow River is considered to be a net remover of sediment from the Loess Plateau^21^,^22^,^23^. However, a recent study^24^ suggests that the Yellow River has provided sediment to the Loess Plateau during the last glacial period, casting doubt on the origins of this climate and atmospheric dust archive. Key to unravelling these questions is constraining the river's past and present sediment sources and dispersal patterns.

Here we constrain these sources and dispersal patterns using the first extensive modern and paleo-river sediment provenance data set based on combined detrital zircon U–Pb dating, heavy mineral and framework petrography. The results show that the Loess Plateau is a major terrestrial sink for Yellow River sediment eroded from the NE Tibetan Plateau.

## Results

### Modern Yellow River provenance data

**Figure 1:**
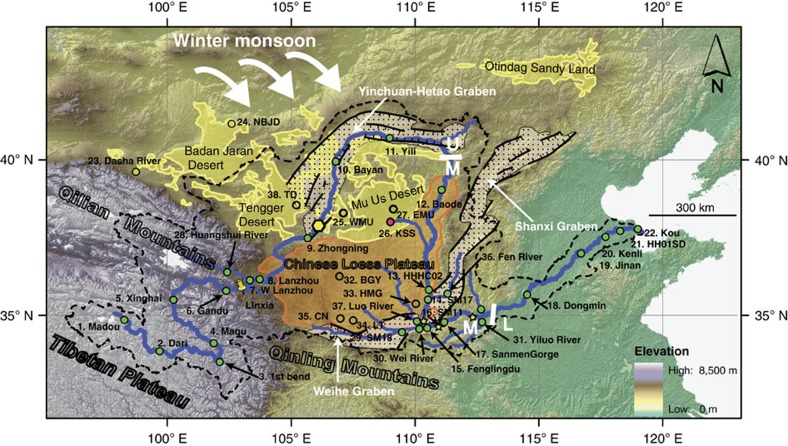
Map showing the Yellow River and the sampling sites. The upper (U), middle (M) and lower (L) reaches of the Yellow River are divided by white lines. The U reach is further subdivided into the plateau/canyon portion and the alluvial platform portion by the Qingtong Gorge (the yellow hexagon with black boundary). Numbers represent locations of provenance samples. Numbers 1–22 represent the main stream sites of the Yellow River. Samples 14 and 14′ are very close, so 14′ is not shown. Location of the Jishi conglomerates is shown with a yellow pin labelled as Linxia. The Wuquan conglomerates and Lanzhou terrace sites in Fig. 2 are near site 8. The M reach paleo-river sites in Fig. 2 are shown with yellow stars. The Yellow River's drainage area is highlighted by thick black dashed contour. The graben sediment filling system^8^ is bounded by black line with ticks filled with dots. Sample description is in Supplementary Table 1.

**Figure 2:**
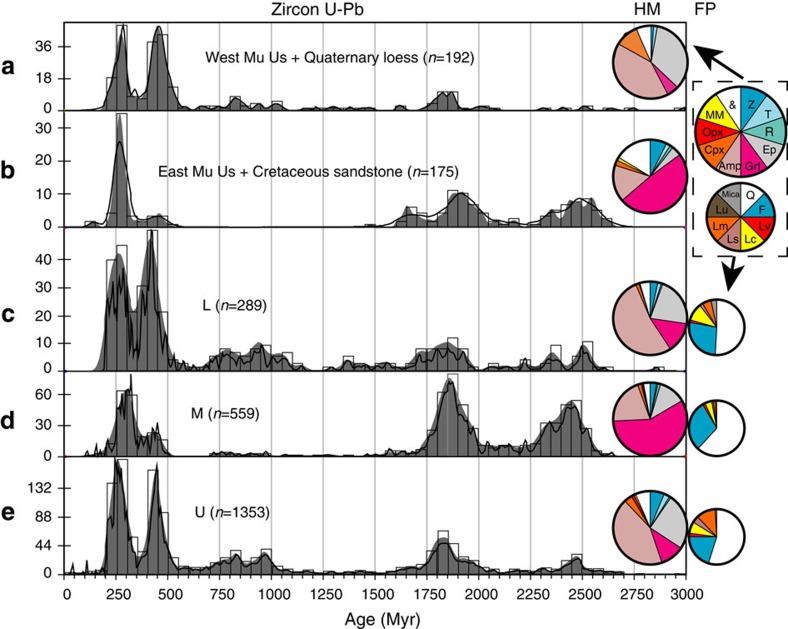
Provenance data from the Yellow River and potential source regions. (**a**) West Mu Us desert and Quaternary loess^24^. (**b**) East Mu Us desert and Cretaceous North China Craton sandstone^24^. (**c**–**e**) Lower (L), middle (M) and upper (U) reach modern Yellow River, respectively. For the zirocn U–Pb age plots, black lines and grey shade are normalized probability density plot (PDP) and kernel density estimation (KDE) plots^67^, respectively, and the open rectangles are age histograms. The big and small wheels inside the dashed rectangle in the upper right corner show the legend of the heavy mineral (HM) and framework petrography (FP) plots, respectively. For HM plots, Z: zircon; T: tourmaline; R: rutile; Ep: epitode; Grt: garnet; Amp: amphibole; Cpx: clinopyroxene; Opx: Orthopyroxene; MM: metasedimentary minerals (chloritoid+staurolite+andalusite+kyanite+sillimanite); &: others. For FP plots: Q: quartz; F: feldspar; L: lithic fragments (Lv: volcanic; Lc: carbonate; Ls: shale/siltstone+chert; Lm: metamorphic; Lu: ultramafic). Provenance data of the individual samples within U (samples 1–11), M (samples 13–17) and L (samples 18–22) are combined for ease of visualization because they have a similar signal within each interval. Individual plots are shown in Supplementary Figs 1 and 2. Zircon U–Pb data are in Supplementary Data sets 1, 2 and 3. Heavy mineral and bulk petrography data are in Supplementary Tables 2 and 3, respectively.

Sediment samples from bars in the upper (Fig. 1; samples 1–11; refer to Supplementary Table 1 for sample information) and lower reaches (samples 18–22) of the modern Yellow River show similar provenance signals (zircon U–Pb data (Supplementary Data sets 1, 2 and 3), heavy mineral (Supplementary Table 2) and bulk petrography (Supplementary Table 3)) and are also similar to the modern western Mu Us desert (sample 25) and the Quaternary Chinese Loess Plateau samples (Fig. 2; Supplementary Figs 1, 2 and 3). In contrast, modern bar sediment samples from the Yellow River middle reach (Fig. 1; samples 12–17) show different signals, similar to the Cretaceous sandstones overlying the North China Craton^24^,^25^ and similar to the modern sands of the eastern Mu Us desert (samples 26 and 27; Fig. 2; Supplementary Figs 1 and 2). The zircon U–Pb ages of the upper and lower reaches samples, and the western Mu Us desert and the Chinese Loess Plateau samples, show two prominent peaks at ∼450 and ∼250 Myr ago, matching NE Tibetan source rock signatures^15^,^24^. In contrast, only one prominent peak at ∼250 Myr ago is expressed in the middle reach, Cretaceous sandstones and the eastern Mu Us desert. In addition, the ages falling between 2,750 and 1,500 Myr ago account for <30% of ages for the upper and lower reaches, the western Mu Us desert and the Chinese Loess Plateau, but comprise >60% of ages from the middle reach, Cretaceous sandstones and eastern Mu Us desert. The heavy mineral assemblages of the upper and lower reaches, western Mu Us desert and Chinese Loess Plateau are dominated by unstable mineral amphibole followed by epidote, while by contrast the middle reach, Cretaceous sandstones and eastern Mu Us desert samples are dominated by stable mineral garnet, with amphibole as the second most abundant mineral type (Fig. 2). In terms of the bulk petrography data, the middle reach has higher quartz content but less lithic fragments than the upper and lower reaches (Fig. 2), indicating higher sediment maturity for the middle reach sediment. This is consistent with their greater garnet content as well as the similarity between heavy mineral and zircon U–Pb signatures of middle reach sediment and the highly weathered Cretaceous sandstones overlying the North China Craton.

### Paleo Yellow River provenance data

**Figure 3:**
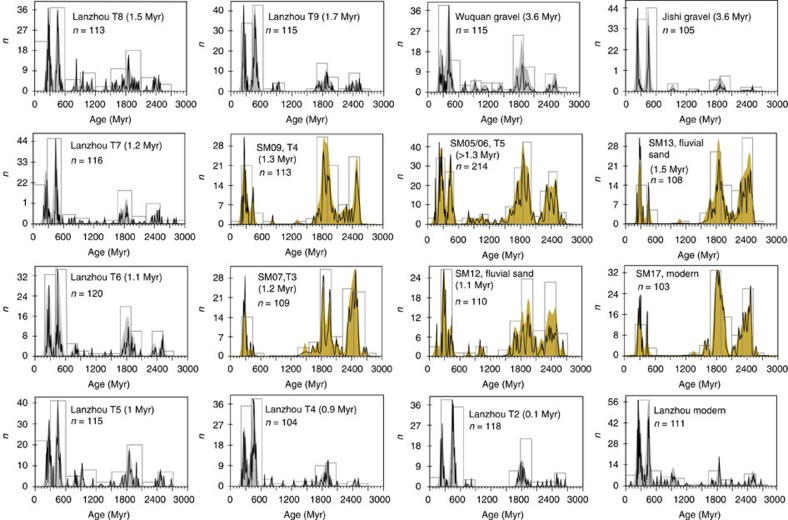
Zircon U–Pb ages of the paleo-upper and -middle reach Yellow River. Upper and middle reach data are in grey and yellow, respectively. Black lines and colour shades are normalized probability density plot (PDP) and kernel density estimation (KDE) plots^67^, respectively, and the open rectangles are age histograms. For quantitative comparison of similarities between these data, please refer to Supplementary Fig. 3. Tx: the terrace number.

Comparison of zircon U–Pb data from paleo-river terrace sediment in the upper reach (near site 8: Lanzhou) with the middle reach (near sites 15 and 16) demonstrates that the situation of the upper and the middle reaches having different provenance persists from at least ∼1.7 Myr ago (Fig. 3; Supplementary Fig. 4). The occurrence of ∼3.6 Myr ago terrace conglomerates in Linxia (Jishi conglomerates; yellow pin in Fig. 1) and Lanzhou (Wuquan conglomerates; site 8) that also show similar zircon U–Pb provenance signatures to the current and Pleistocene upper reach provenance signal (Fig. 3; Supplementary Fig. 4) confirm that the current location and pattern of Yellow River upper reach drainage was broadly formed at least by then.

## Discussion

**Figure 4:**
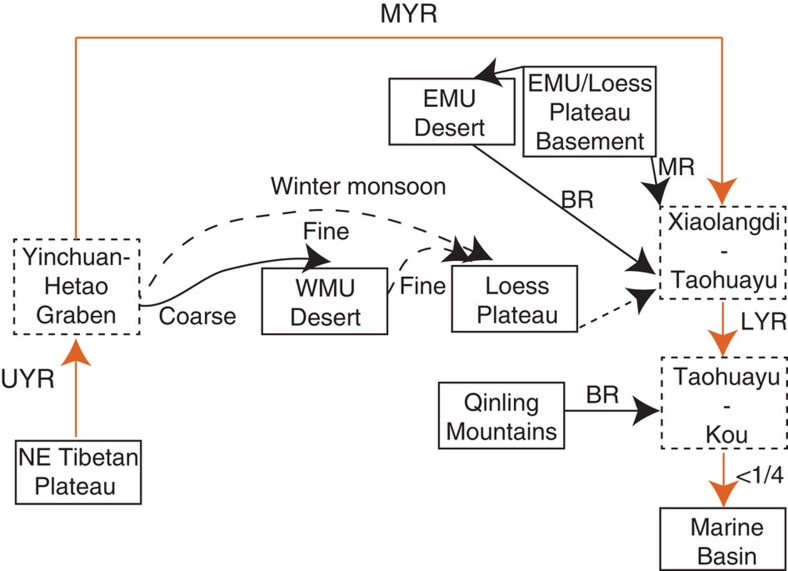
Sediment provenance and dispersal pattern of the Yellow River. The three depositional areas along the Yellow River are shown by dashed rectangles. UYR, upper Yellow River; MYR, middle Yellow River; LYR, lower Yellow River; MR, main river; BR, branch river; EMU, East Mu Us; WMU, West Mu Us. Xiaolangdi is indicated by the yellow star near site 31 in Fig. 1. Taohuayu corresponds to the boundary between the middle and the lower reaches of the Yellow River in Fig. 1. Kou corresponds to site 22 in Fig. 1. River transport and winter monsoon transport are represented by straight and curved lines, respectively. Fine (dashed curved line) and coarse (solid curved line) particles are transported to the Loess Plateau and the western Mu Us desert, respectively, by the East Asian winter monsoon. Less than 1/4 of the lower reach sediment is transported to the marine basin^1^,^30^. The dashed straight line with arrow indicates the relative unimportance of the Loess Plateau in contributing sediment to the middle reach of the Yellow River. The provenance shift in the lower portion of the Yellow River suggests that a new sediment source is introduced and we attribute this source to erosion of the Qinling Mountains by branch rivers.

To understand the significance of these data, we interpret them in the context of the Yellow River drainage. The Yellow River has traditionally been divided into the upper, the middle and the lower reaches, based on geographical position, elevation and erosional/depositional patterns^26^,^27^,^28^. The upper reach and the middle reach each consist of an erosional section and a depositional section (Supplementary Fig. 5), while the lower reach is characterized by sediment deposition alone^26^,^27^,^28^. The upper reach of the Yellow River is subdivided into an erosional plateau/canyon portion and a depositional alluvial plain portion^27^,^28^, separated by the Qingtong Gorge (the yellow hexagon in Fig. 1). Due to the high topographic gradient (Supplementary Fig. 5), the Yellow River flows rapidly and incises in the plateau/canyon portion on the NE Tibetan Plateau, with limited sediment deposition^27^,^28^. When the river passes the Qingtong Gorge, leaves the NE Tibetan Platau and enters the Yinchuan-Hetao Graben system, its velocity decreases resulting in deposition and formation of the Yinchuan-Hetao alluvial platform^8^,^27^ (Fig. 1; Supplementary Fig. 5). Both Quaternary Chinese Loess Plateau sediment and modern western Mu Us sands show similar provenance signatures to this upper reach sediment (Fig. 2) and are located directly downwind of the Yinchuan-Hetao Graben system under East Asian winter monsoon dust transportation circulation (Fig. 1). Thus, we propose that these extensive river sediment deposits serve as a major source for the western Mu Us desert and the Loess Plateau. By contrast, the middle reach of the Yellow River is dominantly characterized by erosion^8^,^27^, as the river enters the Jinshan Canyon (from the upper/middle reach boundary to site 15; Supplementary Fig. 5). A small depositional zone^27^ occurs at the very end of the middle reach between Xiaolangdi (the yellow star near site 31 in Fig. 1) and Taohuayu (the boundary between the middle and the lower reaches in Fig. 1). The erosional portion of the middle reach has formed deeply incised canyons into Cretaceous sandstone bedrock and underlying North China Craton^8^,^25^,^29^, which, as shown in the provenance data (Fig. 2), is the dominant river sediment source in the middle reach, rather than the overlying loess^30^. We propose a conceptual model for Yellow River sediment dynamics and Chinese Loess Plateau formation in which both the western Mu Us desert and the Loess Plateau materials are sourced from Yellow River alluvium that is eroded and transported from the NE Tibetan Plateau, deposited in Yinchuan-Hetao alluvial platform, and is then locally redistributed by winter monsoon winds (Fig. 4).

**Figure 5:**
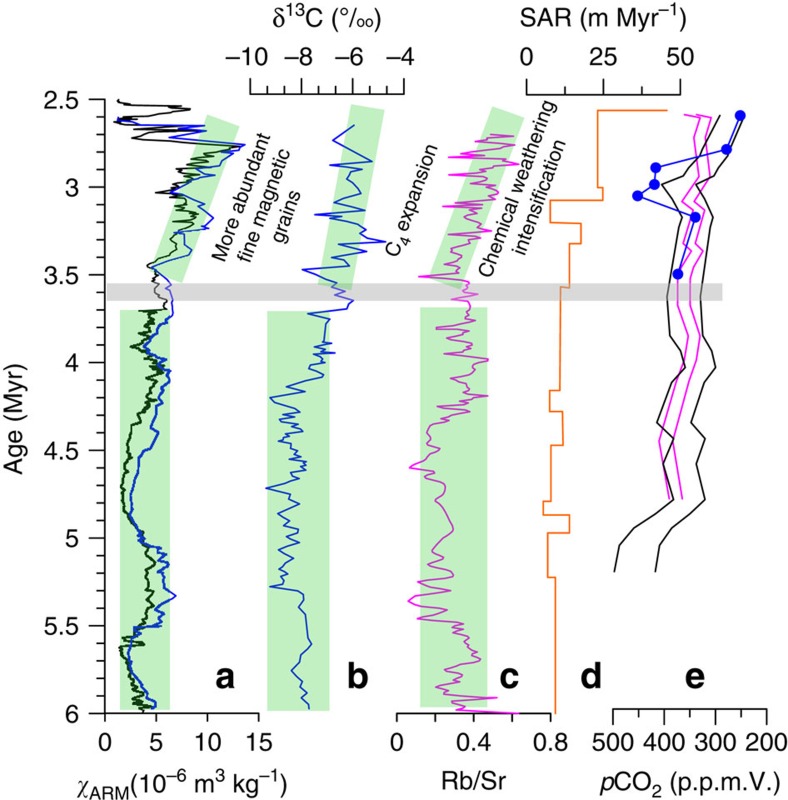
Paleoclimatic records during 6-2.5 Myr. (**a**–**c**) Loess magnetic^55^, carbon isotope^54^ and Rb/Sr ratio^33^ records of the East Asian summer monsoon variations. (**d**) Sedimentation accumulation rate (SAR) from the central Chinese Loess Plateau^40^. (**e**) Recent atmospheric *p*CO_2_ records^68^,^69^. *χ*_ARM_, anhysteretic remanent magnetization susceptibility. Maximum and minimum ranges of alkenone-based CO_2_ data are shown with black (Ocean Drilling Project Site 999, Caribbean Sea) and pink (Ocean Drilling Project Site 925, western Atlantic Ocean) lines; the boron-based CO_2_ data trend is shown by the blue line with dots (Ocean Drilling Project Site 999). We note that ref. 69 presented six alkenone-based CO_2_ records for the Plio-Pleistocene period and that they all show a decreasing trend. However, site 925 has the highest precision and does not show anomalously high CO_2_ after 2.7 Myr ago, as in site 806. As such, site 925 data were selected for inclusion here.

While some past research emphasizes aeolian transport from the Chinese northern deserts in formation of the Chinese Loess Plateau^31^,^32^,^33^,^34^,^35^,^36^, the evidence for this is also compatible with our conceptual model (Figs 1 and 4). Loess grain size has a southward decreasing trend^34^, which is consistent both with a northern Chinese deserts or Yellow River source for the loess. However, our data show that sands of the western Mu Us are also derived from the Yellow River. Furthermore, recent desert drilling^37^,^38^ has demonstrated a late Pleistocene formation age (∼1 Myr ago) for the Tengger and the Badan Jaran desert (Fig. 1). This is significantly younger than the formation age of the Chinese loess and suggests that direct dust transport from these two deserts is only a minor factor in Chinese Loess Plateau formation. In contrast, loess^39^ immediately south of the Mu Us desert (Jingbian; south of site 26 at the current boundary of the Loess Plateau) has a basal age of 3.5 Myr ago, synchronous with increased sedimentation rate across the central Loess Plateau generally^40^,^41^ (Fig. 5) and with the earliest terrace deposits from the river. Furthermore, available satellite imagery^42^ from a storm event (14–17 April 1998) clearly shows that modern dust storms travelling over the Loess Plateau originate in areas north to northwest of this region, including the Yinchuan-Hetao floodplain. The Yinchuan-Hetao floodplain is, along with the Mu Us, the last major possible sediment source for these storms before they reach the Loess Plateau, providing modern observations consistent with our conceptual model.

The coarse grain size of the Mu Us desert sands makes the possibility of direct aeolian transport from NE Tibetan source regions unlikely, therefore, requiring fluvial transport followed by only more localized aeolian transport. Furthermore, the abrupt shift in provenance away from loess signatures in the middle reach (Fig. 2) occurs precisely when eroding loess on the Loess Plateau would be expected to overwhelm the sediment budget of the river (Figs 1 and 2). We discount dilution as an explanation for this shift in provenance signals (Fig. 2) away from loess in the middle reach as this would require orders of magnitude increases in sediment load to explain the change from the double peak dominance (∼450 and ∼250 Myr ago) in the loess and upper reach zircon U–Pb data, to the ∼250 Myr ago single peak dominance at Baode (sample 12) (Fig. 2; Supplementary Figs 1 and 6). Sediment load only increases ∼24% from the end of the upper reach to Baode^21^. Thus, we argue that the Loess Plateau and adjacent western Mu Us desert, where provenance signatures match the upper reaches' and demonstrate a NE Tibet origin^24^ (Fig. 2), are acting as sinks for NE Tibetan Plateau-derived sediment carried in the upper reach of the Yellow River.

As an approximate check on the feasibility of our model, we calculate the first-order length of time required for Yellow River sediment to fill the Quaternary portion of the approximate volume of the Loess Plateau, using the modern sediment load measured at the Xunhua observation station (∼20 km east of site 6). The amount of time (∼1.65 Myr ago) is of the same order as the basal age of the Quaternary, suggesting Yellow River sediment flux is sufficient to explain the Loess Plateau volume, consistent with our model (Methods). There are considerable uncertainties on this estimate, especially over land use changes and changes in river sediment load through time, as well as possible erosion or deflation on the Loess Plateau. Prior research^43^ suggests that moderate land use by humans may increase sediment yield by a factor of 2–3. As such, even if we suggest a 2–5 factor decrease in sediment load of the Yellow River when there was no significant human activity, the calculated time required to fill in the Quaternary portion of the Loess Plateau (3.3–8.3 Myr ago) is still on the same order as the bottom age of the Quaternary. However, given these unavoidable uncertainties, we stress that we only use this calculation to determine whether our hypothesis is generally plausible (that is, that the required time is of the order of millions of years rather than 10–100 s of millions of years). Thus, it seems feasible that the large increases in loess sediment accumulation rate and area observed during the Pliocene and Pleistocene^40^ (Fig. 5d) at least partially result from increased river incision and downstream transport of material from NE Tibet via the Yellow River, rather than due solely to intensified aridity as previously suggested^44^.

It is interesting to note that the provenance of the lower reach Yellow River is similar to that of the upper reach after the confluence with the Yiluo River (Fig. 1). The provenance shift appears to indicate the effects of the Yiluo River (Fig. 1), which brings in sediment derived from the Qinling Mountains characterized by abundant Phanerozoic zircon U–Pb ages with a double peak^45^,^46^,^47^ at 450 and 250 Myr ago, resulting in a similar signal between the lower and upper reaches, despite different source admixtures (Figs 1 and 4). Again, this provenance shift is consistent with modern observation that the middle reach eroded sediment is deposited between Xiaolangdi and Taohuayu^27^,^28^, providing further evidence for terrestrial storage of denudation materials for large rivers. For the modern Yellow River, it is well recorded that only 24% of the sediments flowing past the Sanmen Gorge enter the ocean and the rest are deposited on alluvial plain and the delta regions^1^,^30^, consistent with our model.

These findings that the NE Tibet-derived sediments of the upper reach are stored on the Loess Plateau and the western Mu Us desert after 3.6 Myr ago requires a fundamental change in our understanding of the origins of Chinese loess dust and the impact of the Yellow River on the Chinese Loess Plateau. Furthermore, it means that orogenic hinterland erosional signals can be masked in marine sedimentary records by sediment storage in terrestrial basins. If the Tibetan Plateau experienced a phase of northeastward growth and accelerated denudation during the late Pliocene, as suggested by climate and tectonic research^11^,^33^,^48^,^49^, a significant proportion of the denuded sediment would have been stored on land instead of in the adjacent marine basin. Indeed, this accelerated denudation is reflected in increases in loess sediment accumulation rate observed during the Pliocene and Pleistocene^40^ (Fig. 5d). Our findings mean that it is unlikely that marine records will properly detect this event. Unfortunately, there are no Plio-Pleistocene provenance data from the Bohai Sea, where the Yellow River drains, to test this and there are a range of possible responses of the marine sediment record to the terrestrial storage demonstrated here. Heavy mineral data from modern Bohai Sea surface sediment^50^ show that Yellow River marine input is characterized by an assemblage similar to our lower reaches data (for example, dominated by amphibole), consistent with our model. However, there is a clear need for systematic provenance analysis of Plio-Pleistocene Bohai Sea sediment to determine the marine response to NE Tibetan denudation in light of the terrestrial sediment storage that our data demonstrate.

The Yellow River may not be unique in storing most of its upstream-eroded materials on land. Sediment budget research widely demonstrates that although large rivers drain orogenic belts, the majority of the eroded sediments are stored in terrestrial basins and trailing edge margins^51^,^52^,^53^. Instead, small mountain rivers, which are often close to steep active continental margins, play a key role in transporting materials to the ocean^52^. Thus, our model can help explain the recent evidence for mismatches between the terrestrial sedimentation rate record and the marine Beryllium isotope proxy record of denudation, as well as between late Cenozoic global CO_2_ and marine sediment volume records^3^.

The increased NE Tibetan denudation and apparent onset of enhanced Yellow River drainage at ∼3.6 Myr ago are coincident with an increase in C_4_ plant proportion^54^, degree of chemical weathering^33^ and pedogenic magnetic mineral concentration^55^ (Fig. 5), suggesting that an enhanced monsoon climatic threshold was also reached at the same time. However, our data do not allow us to determine whether this monsoon increase and concurrent increased climate fluctuation amplitude^9^ or rather tectonic uplift^33^,^48^ caused this increased denudation in NE Tibet. While these enhanced summer monsoon conditions could have promoted increased drainage and erosion, coincident with increased loess accumulation rate on land, the relationship between climate change, Tibetan uplift, and denudation has always been difficult to determine^56^,^57^,^58^. Despite this, one potential inference from this result is that the increased NE Tibetan Plateau Pliocene denudation recorded in the loess deposits may be an important driver for Pliocene climate cooling. Increased denudation is known to increase terrestrial chemical weathering and organic carbon burial^10^,^59^ and may have also increased the flux of dust to the Pacific ocean, stimulating marine phytoplankton production^60^,^61^. All of these factors promote Pliocene atmospheric CO_2_ drawdown (Fig. 5e) and climatic cooling.

In summary, our research provides the first comprehensive provenance data set demonstrating that the majority of NE Tibet denuded material was deposited on the Loess Plateau and the western Mu Us desert, instead of being effectively delivered to the lower reach and the marine basins since at least the middle Pleistocene. This not only casts new light on the origins of Chinese loess but it undermines the principle of using marine sediment to infer terrestrial denudation and to understand the complex relationship between denudation and climate change. Furthermore, our data suggest that increased NE Tibet denudation recorded in Yellow River-derived sediment on the Loess Plateau is a potentially important driver in Pliocene climate cooling.

## Methods

### Framework petrography

Samples were collected from active fluvial bars of the Yellow River (Huang He) and some of its major tributaries. They were impregnated with Araldite, cut into standard thin sections, stained with alizarine red to distinguish dolomite and calcite and analysed by counting 400 points under the microscope (Gazzi-Dickinson method^62^). Sands were classified according to their main components (Q=quartz; F=feldspars; L=lithic fragments), considered only where exceeding 10% QFL and listed in order of abundance (for example, in a litho-feldspatho-quartzose sand Q>F>L>10% QFL). Full quantitative information was collected on coarse-grained rock fragments, and metamorphic types were classified according to protolith composition and metamorphic rank. Very-low- to low-rank metamorphic lithics, for which protoliths can still be inferred, are subdivided into metasedimentary (Lms) and metavolcanic (Lmv) categories. Medium- to high-rank metamorphic lithics are subdivided into felsic (metapelite, metapsammite and metafelsite; Lmf) and mafic (metabasite; Lmb) categories. Median grain size was determined in thin section by ranking and visual comparison with sieved standards.

### Heavy minerals

Heavy minerals were separated by centrifuging in sodium polytungstate (density ∼2.90 g cm^−3^), and recovered by partial freezing with liquid nitrogen. The obtained fraction was weighted and mounted for counting on glass slides with Canada balsam. On grain mounts, between 200 and 250 transparent heavy mineral grains were point-counted at suitable regular spacing under a petrographic microscope to obtain real volume percentages^63^.

### Zircon U–Pb dating

Detrital zircon grains were separated by standard heavy liquid techniques, selected randomly and analysed by laser ablation inductively coupled plasma mass spectrometry in the Department of Geological Sciences at the University of Texas at Austin (seven Lanzhou terrace samples, one Wuquan conglomerate sample and three upper reach modern river samples: 8, 10 and 11), University of Arizona (Linxia gravel sample) and University College London (the rest of the modern Yellow River samples), following the standard procedure of each laboratory^16^,^64^,^24^. We apply a 15–10% discordance filter to the generated data. For ages younger than 1,000 Myr ago, the discordance is defined as (^207^Pb/^235^U–^206^Pb/^238^U)/ ^207^Pb/^235^U*100; for ages older than 1,000 Myr ago, the discordance is defined as (^207^Pb/^206^Pb–^206^Pb/^238^U)/ ^207^Pb/^206^Pb*100. ^206^Pb/^238^U ages were adopted for the ages younger than 1,000 Myr ago, while ^207^Pb/^206^Pb ages were adopted for the ages older than 1,000 Myr ago, although we slightly shift the cutoff age so as to not break cluster ages for different samples.

### Mass balance calculation

We calculate the approximate, first-order amount of time required for Yellow River sediment to fill the Quaternary portion of the Loess Plateau using the modern sediment load data in Xunhua station (∼20 km east of site 6). The approximate timing (1.65 Myr ago) is of the same order as the basal age of the Quaternary, fully consistent with our model. The Loess Plateau area^31^ (A) is set to 4.4 × 10^11^ m^2^. Loess thickness (T) is set to 100 m (ranging from 200 to 0 m from west to east, respectively, during the Quaternary). Dry density^65^ of loess (D) is set to 1,500 kg m^−3^. Annual sand transport amount (AA) in the Xunhua station^66^ (year 1946–1985; before the dam construction) is 4 × 10^7^ ton per year.


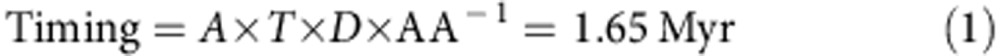


## Additional information

**How to cite this article:** Nie, J. *et al*. Loess Plateau storage of Northeastern Tibetan Plateau-derived Yellow River sediment. *Nat. Commun.* 6:8511 doi: 10.1038/ncomms9511 (2015).

## Supplementary Material

Supplementary Information Supplementary Figures 1-6, Supplementary Tables 1-3 and Supplementary References

Supplementary Dataset 1 Zircon U-Pb data generated at the University College London

Supplementary Dataset 2 Zircon U-Pb data generated at the University of Texas in Austin

Supplementary Dataset 3 Zircon U-Pb ages generated at the University of Arizona
