# Corrigendum: Loess Plateau storage of Northeastern Tibetan Plateau-derived Yellow River sediment

**DOI:** 10.1038/ncomms10831

**Published:** 2016-02-18

**Authors:** Junsheng Nie, Thomas Stevens, Martin Rittner, Daniel Stockli, Eduardo Garzanti, Mara Limonta, Anna Bird, Sergio Andò, Pieter Vermeesch, Joel Saylor, Huayu Lu, Daniel Breecker, Xiaofei Hu, Shanpin Liu, Alberto Resentini, Giovanni Vezzoli, Wenbin Peng, Andrew Carter, Shunchuan Ji, Baotian Pan

Nature Communications 6: Article number: 851110.1038/ncomms9511 (2015); Published: 10092015; Updated: 02182016.

The original version of this Article contained errors in the Supplementary Information files: Zircon U-Pb age results for sample 23, shown in Supplementary Fig. 3, are incorrect, and missing from Supplementary Data 1, while several identification labels relating to Yellow River Lanzhou terraces samples are missing from Supplementary Data 2. Supplementary Data 1 and 2 have now been updated to provide the missing information, while the corrected version of Supplementary Fig. 3 appears below.


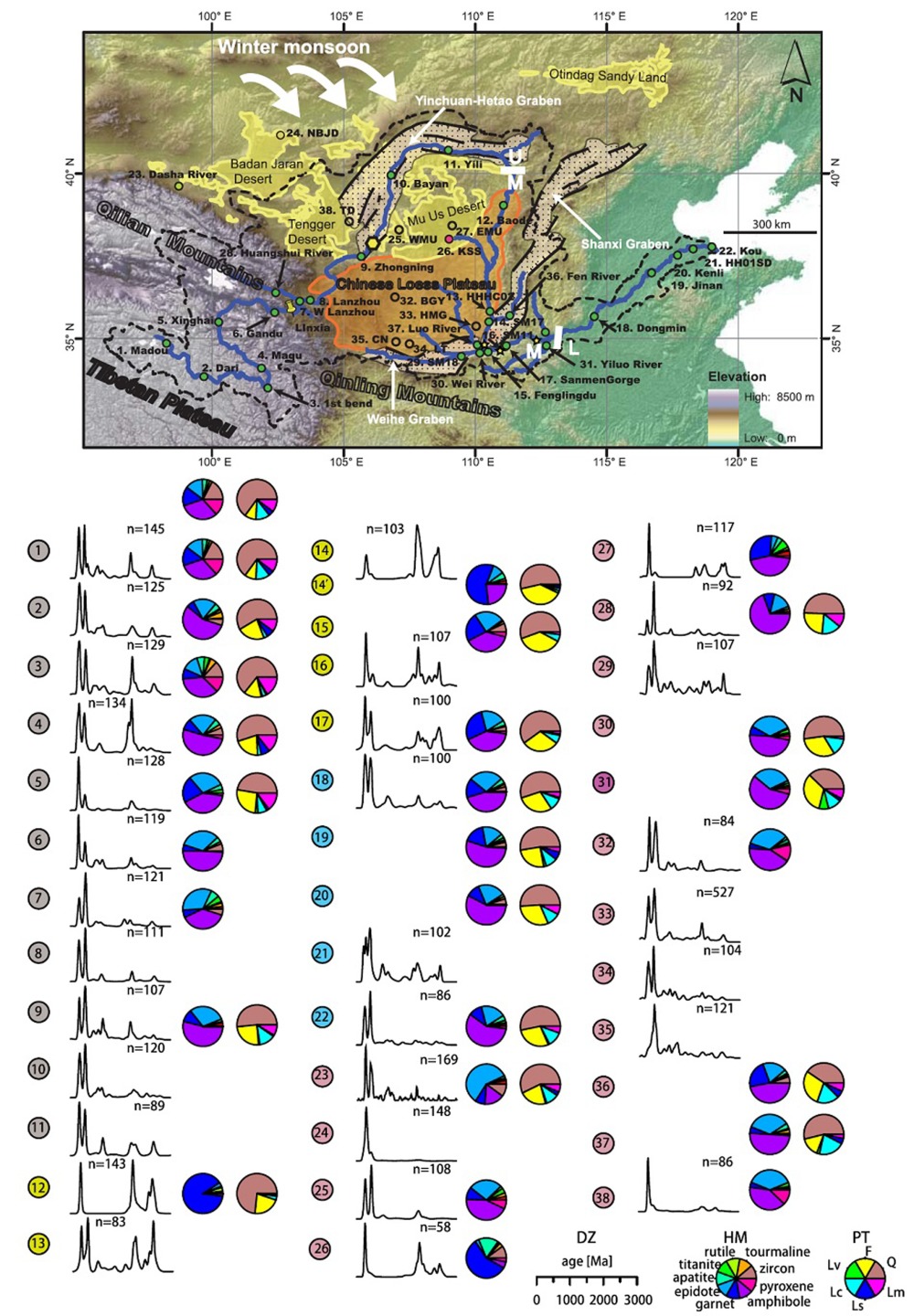


**Supplementary Figure 3**

