# Supplementary material for: Loess Plateau storage of Northeastern Tibetan Plateau-derived Yellow River sediment
Source: Nat Commun. 2015 Oct 9;6:8511. doi: 10.1038/ncomms9511 (PMC4633828; doi:10.1038/ncomms9511)
Supplement: Supplementary Information — Supplementary Figures 1-6, Supplementary Tables 1-3 and Supplementary References [file ncomms9511-s1.pdf]

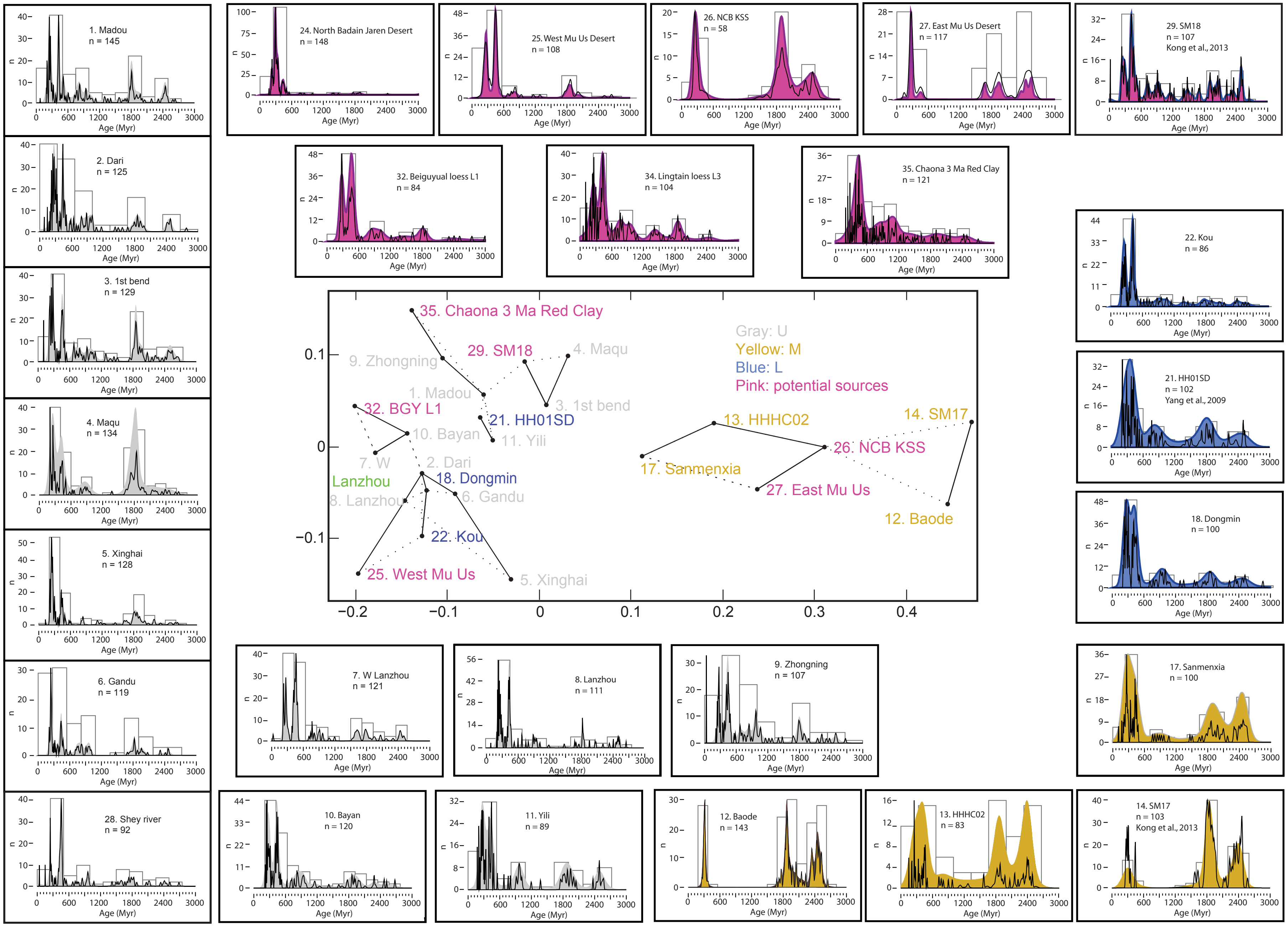
1. Madou
n = 145
2. Dari
n = 125
3. 1st bend
n = 129
4. Maqu
n = 134
5. Xinghai
n = 128
6. Gandu
n = 119
28. Shey river
n = 92
24. North Badain Jaren Desert
n = 148
25. West Mu Us Desert
n = 108
26. NCB KSS
n = 58
27. East Mu Us Desert
n = 117
29. SM18
n = 107
Kong et al., 2013
32. Beiguyual loess L1
n = 84
34. Lingtain loess L3
n = 104
35. Chaona 3 Ma Red Clay
n = 121
22. Kou
n = 86
21. HH01SD
n = 102
Yang et al., 2009
18. Dongmin
n = 100
17. Sanmenxia
n = 100
7. W Lanzhou
n = 121
8. Lanzhou
n = 111
9. Zhongning
n = 107
10. Bayan
n = 120
11. Yili
n = 89
12. Baode
n = 143
13. HHHC02
n = 83
14. SM17
n = 103
Kong et al., 2013
Age (Myr)
n
Gray: U
Yellow: M
Blue: L
Pink: potential sources
35. Chaona 3 Ma Red Clay
29. SM18
4. Maqu
9. Zhongning
1. Madou
32. BGY L1
3. 1st bend
21. HH01SD
10. Bayan
11. Yili
7. W
Lanzhou
2. Dari
18. Dongmin
8. Lanzhou
6. Gandu
22. Kou
25. West Mu Us
5. Xinghai
13. HHHC02
14. SM17
26. NCB KSS
17. Sanmenxia
27. East Mu Us
12. Baode

Supplementary Figure 1 Detrital zircon U-Pb ages from modern Yellow River sediment. Zircon U-Pb ages of example loess (32, 34), red clay (35), North China Craton Cretaceous sandstone (26) and deserts (24, 25, 27), and Wei River (29) are also shown. Black lines and shaded areas are normalized probability density plot (PDP) and Kernel Density Estimation (KDE) plots[1], respectively, and the open rectangles are age histograms. We note that the age pattern is independent of grain size analyzed (Supplementary Figure 6) and that the middle reach data consistently exhibits more abundant Paleoproterozoic ages (~2500-1500 Myr) than the upper reach data. The middle area shows a non-metric multi-dimensional scaling (MDS) plot[2] of representative samples, showing quantitative comparison of similarities. Solid lines mark the closest neighbors and dashed lines the second closest neighbors. For sample information, please refer to Figure 1 and Supplementary Table 1.

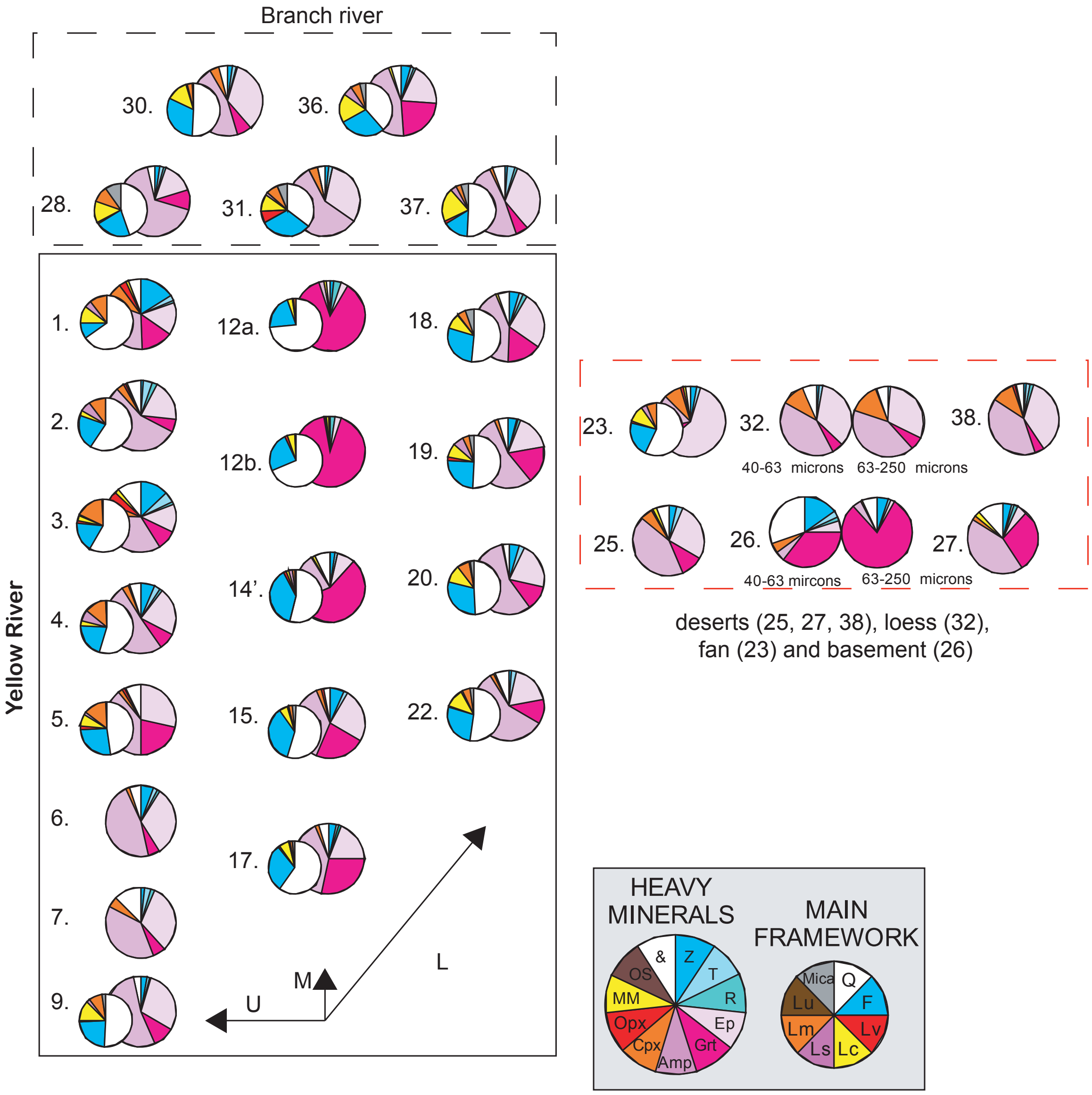

Supplementary Figure 2 Heavy mineral and bulk petrography data from modern Yellow River sediment and potential source regions (deserts, basement, loess and fan). Z: zircon; T: tourmaline; R: rutile; EP: epitode; Grt: garnet; Amp: amphibole; Cpx: clinopyroxene; Opx: Orthopyroxene; MM: metasedimentary minerals (chloritoid + staurolite + andalusite + kyanite + sillimanite). Q: quartz; F: feldspar; L: lithic fragments (Lv: volcanic; Lc: carbonate; Ls: shale/siltstone + chert; Lm: metamorphic; Lu: ultramafic). Sample 12 was analyzed twice [12a (200 grains) and 12b (206 grains)] to test the robustness of the results. We also use a R package designed by Pieter Vermeesch to present individual zircon U-Pb Kernel Density Estimation (KDE) plots[1] and heavy mineral/ bulk petrography pie charts side by side to promote easier visualization (Supplementary Figure 3). For sample information, please refer to Figure 1 and Supplementary Table 1.

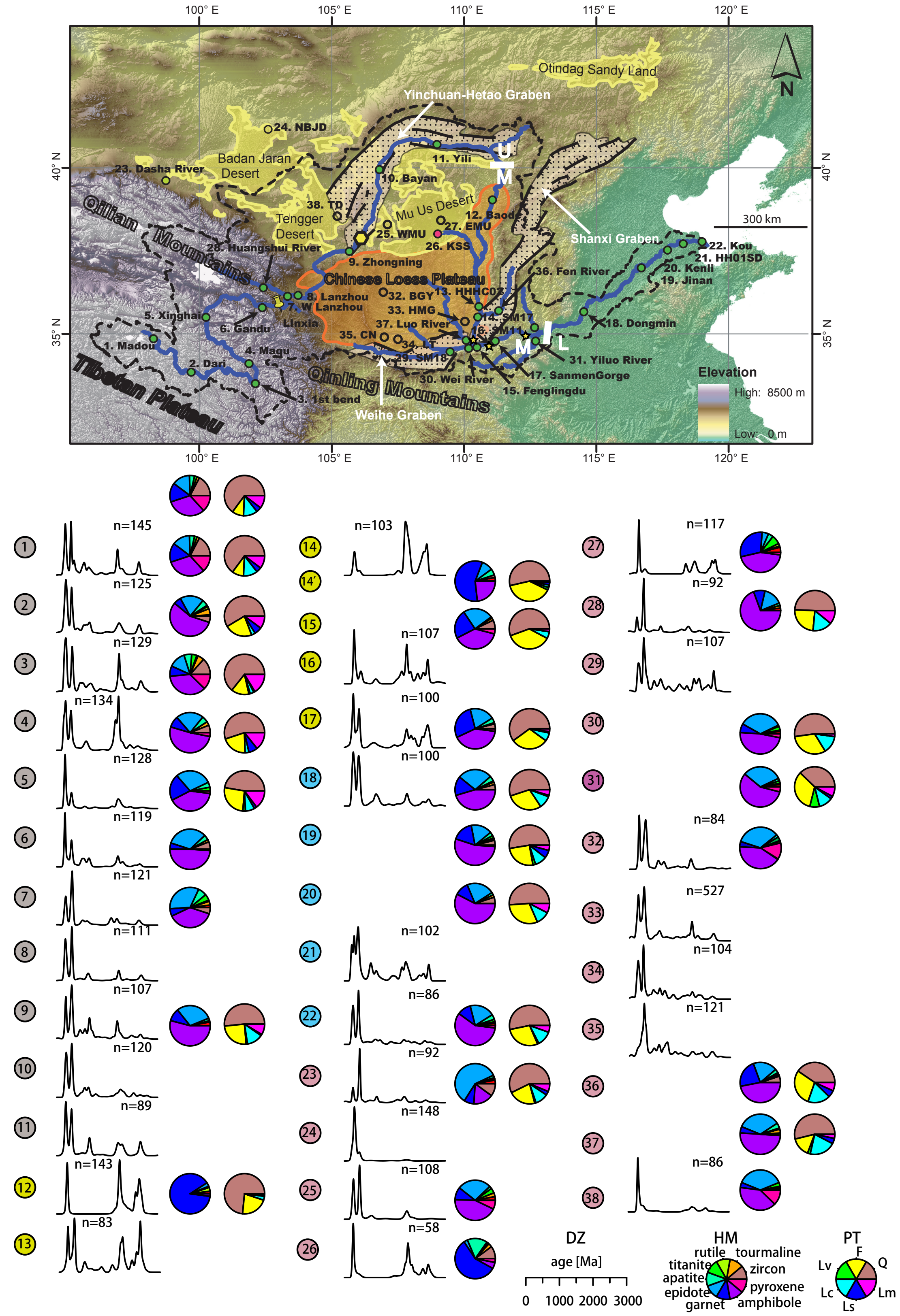

Supplementary Figure 3 Summary plot of all provenance data listed in Supplementary Table 1. Top: Location map. Bottom: Plot of the detrital zircon (DZ) U-Pb Kernel Density Estimation[1,3] (KDE; left), heavy mineral compositions (HM; middle) and framework petrography (PT; right). n = the number of zircon grains analyzed. Numbers in circle correspond to those in Supplementary Table 1. Gray number: upper reach; yellow number: middle reach; blue number: lower reach; pink number: non-Yellow River samples.

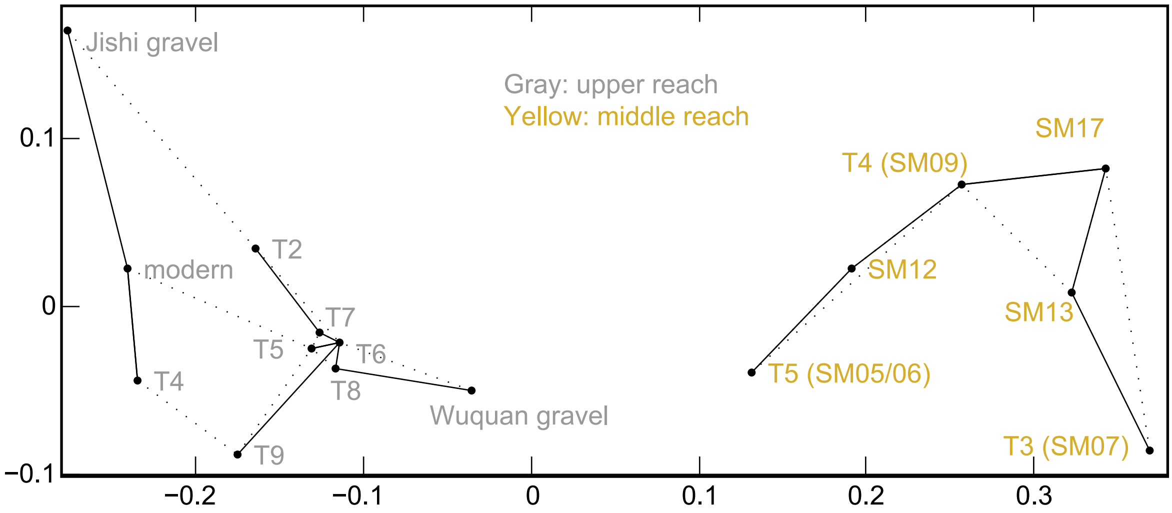

Supplementary Figure 4 Non-metric multi-dimensional scaling (MDS) plots[2] of samples in Figure 3, showing quantitative comparison of similarities between these data. Solid lines mark the closest neighbors and dashed lines the second closest neighbors.

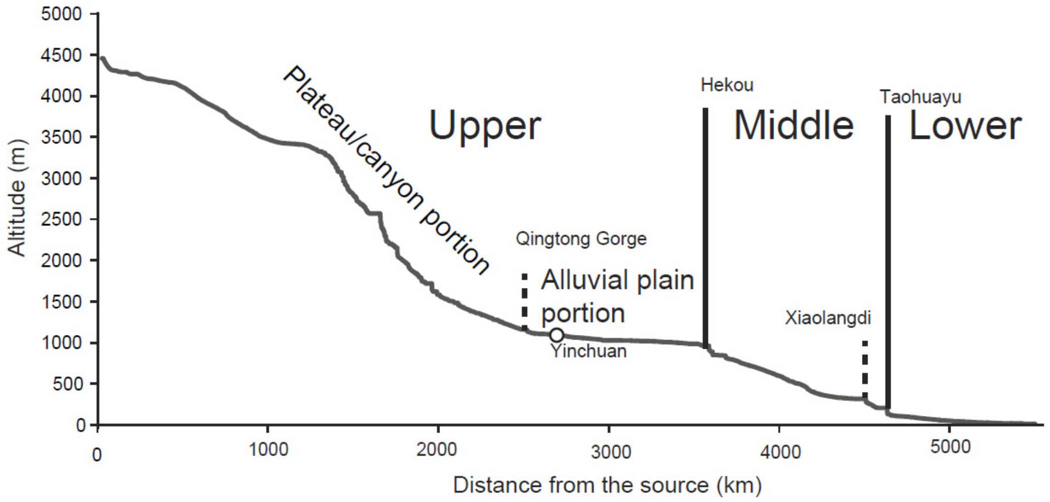

Supplementary Figure 5 The longitudinal profile of the Yellow River. The solid lines divide the river into the upper, middle and lower reaches. The dashed line at Qingtong Gorge divides the upper reach into the plateau/canyon portion and the alluvial plain portion. The dashed line at Xiaolangdi divides the middle reach into an erosional section and a depositional section. We use a digital elevation model (DEM) with a resolution of 180 m resampled from Shuttle Radar Topography Mission (SRTM) 90m DEM. The procedure followed the instructions from http://www.geomorphtools.org/index.htm.

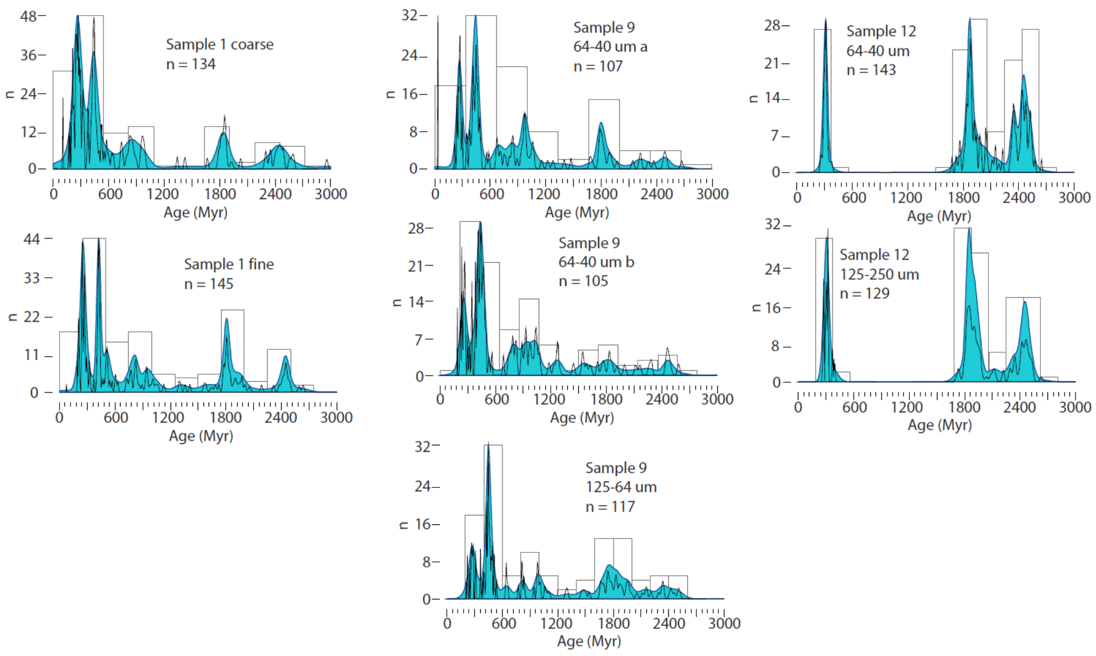

Supplementary Figure 6 Detrital zircon U-Pb age distribution pattern comparison for different grain sizes. Black lines and shaded areas are normalized probability density plot (PDP) and Kernel Density Estimation (KDE) plots [1] respectively, and the open rectangles are age histograms. For sample 1 'coarse' and 'fine' refer to sediment characteristics estimated in the field. For other samples the measured size fraction is indicated.

Supplementary Table 1 Summary table for provenance samples used in this study

Sample#	Latitude	Longitude	Description	Sample name	U-Pb	HM	FP	Reference
1	N34° 53' 7.92"	E098° 10' 13.08"	YR fluvial sand, upper reach	CH12-17	Yes	Yes	Yes	this study
2	N33° 47' 49.08"	E099° 41' 43.14"	YR fluvial sand, upper reach	CH12-18	Yes	Yes	Yes	this study
3	N33° 31' 57.48"	E102° 27' 34.02"	YR fluvial sand, upper reach	CH12-19	Yes	Yes	Yes	this study
4	N33° 57 ' 32.52"	E102° 05' 13.38"	YR fluvial sand, upper reach	CH12-20	Yes	Yes	Yes	this study
5	N35° 30' 5.7"	E100° 10' 1.98"	YR fluvial sand, upper reach	CH12-16	Yes	Yes	Yes	this study
6	N35° 52' 45"	E102° 13' 33"	YR fluvial sand, upper reach	CH12-21	Yes	Yes	No	this study
7	N36° 08' 22.14"	E103° 36' 32.7"	YR fluvial sand, upper reach	YR-1	Yes	Yes	No	this study
8	N36° 04' 52.1"	E103° 51 '51.8"	YR fluvial sand, upper reach	YR-9C	Yes	No	No	this study
9	N37° 31' 19.08"	E105° 40' 00"	YR fluvial sand, upper reach	CH11YR03-04	Yes	Yes	Yes	this study
10	N39° 55' 28.98"	E106° 43' 25.74"	YR fluvial sand, upper reach	BY	Yes	No	No	this study
11	N40° 35' 10.12"	E108° 46' 11.82"	YR fluvial sand, upper reach	YL	Yes	No	No	this study
12	N39° 00' 45.48"	E111° 01' 18.18"	YR fluvial sand, middle reach	CH11YR02	Yes	Yes	Yes	this study
13	N35° 24' 30"	E110° 27' 19.5"	YR fluvial sand, middle reach	HHHC02	Yes	No	No	4
14	very close to 14'		YR fluvial sand, middle reach	SM17	Yes	No	No	5
14'	N35°39' 26.35 "	E110°35' 49.69"	YR fluvial sand, middle reach	S4511	no	Yes	Yes	this study
15	N34° 36' 40.56"	E110° 19' 31.37"	YR fluvial sand, middle reach	S4509	no	Yes	Yes	this study
16			YR fluvial sand, middle reach	SM11	Yes	No	No	5
17	N34° 47' 33.2"	E111° 08' 10.7"	YR fluvial sand, middle reach	S4512	Yes	Yes	Yes	this study
18	N35° 25' 38.8"	E115° 06' 54.7"	YR fluvial sand, lower reach	S4517A	Yes	Yes	Yes	this study
19	N36° 43' 35.7"	E116° 59' 22.7"	YR fluvial sand, lower reach	S4521	No	Yes	Yes	this study
20	N37° 36' 13.27"	E118° 31' 55.34"	YR fluvial sand, lower reach	S4518	No	Yes	Yes	this study
21	N37° 36' 34.74"	E118° 23' 30.42"	YR fluvial sand, lower reach	HH01SD	Yes	No	No	4
22	N37° 45' 38.09"	E119° 09' 20.95"	YR fluvial sand, lower reach	S4520	Yes	Yes	Yes	this study
23	N39° 17' 6.42"	E100° 12' 1.68"	Qilian fan	CH12-05	Yes	Yes	Yes	this study
24	N41° 57' 7.2"	E102° 16' 25.38"	N Badan Jaran desert	CH12-10	Yes	No	No	this study
25	N38° 29' 32.22"	E107° 13' 37.08"	Western Mu Us Desert	MD-9	Yes	Yes	No	6
26	N37° 59' 31.86"	E108° 52' 12.18"	Cretaceous sandstone in Mu Us deser	MD-6	Yes	Yes	No	6
27	N38° 28' 36.54"	E108° 45' 45.84"	Eastern Mu Us desert	MD-2	Yes	Yes	No	6
28	N36° 28' 43.44"	E102° 19' 19.02"	Huangshui River, Branch river of the Y	CH12-15	Yes	Yes	Yes	this study
29			Wei River, Branch river of the YR	SM18	Yes	No	No	5
30	N34° 39' 45.10"	E110° 07' 43.34"	Wei River, Branch river of the YR	S4507	No	Yes	Yes	this study
31	N34° 48' 21.6"	E113° 02' 58.7"	Yiluo River, Branch river of the YR	S4516	No	Yes	Yes	this study
32	N36° 37' 21.30"	E107° 17' 12.18"	Beiguoyuan loess L1	CH04116-19	Yes	Yes	No	6
33	N35° 43' 33.18"	E109° 03' 58.7"	Heimugou loess (L1-L33)	HMG	Yes	No	No	7
34	N43° 58' 50.34"	E107° 33' 32.28"	Lingtai loess L3	CH1106-03	Yes	No	No	this study
35	N35° 02' 23.7"	E107° 13' 34.02"	Chaona red clay of 3 Ma	CN-3Ma	Yes	No	No	8
36	N35° 34' 00.28"	E110° 44' 55.65"	Fen River, Branch river of the YR	S4510	No	Yes	Yes	this study
37	N34° 41' 27.98"	E110° 08' 19.80"	Luo River, Branch river of the YR	S4508	No	Yes	Yes	this study
38	N38° 35' 23.94"	E105° 28' 40.2"	Tengger desert	Td-1	Yes	Yes	No	this study

YR: Yellow River; HM: heavy mineral; FP: framework petrography

Supplementary Table 2 Heavy mineral counts for samples in this study. For sample information, please refer to Figure 1 and Supplementary Table 1.

Sample #	zircon	tourmaline	rutile	Ti oxides	titanite	apatite	monazite	others	epidote	prehnite	garnet	chloritoid	staurolite	andalusite	kyanite	sillimanite	amphibole	clinopyroxene	orthopyroxene	olivine	spinel
1	17	2	0	0	2	4	0	0	14	0	15	1	0	0	0	0	31	9	3	0	0
2	1	5	2	0	1	5	0	0	19	0	6	0	0	0	0	1	56	4	0	0	0
3	13	5	1	0	4	6	0	0	13	0	8	0	0	2	0	0	35	5	6	0	0
4	6	3	0	0	0	5	0	0	22	0	9	0	0	0	0	0	51	2	0	0	0
5	0	0	0	0	3	4	0	0	29	0	21	0	0	0	0	0	40	1	0	0	0
6	6	2	0	0	0	4	0	0	33	0	5	0	0	0	0	0	49	1	0	0	0
7	2	3	1	0	5	7	0	1	33	0	6	0	0	0	0	0	38	5	0	0	0
9	3	1	0	0	0	2	0	0	29	0	10	0	0	0	0	0	53	0	0	0	0
12	2	0	3	0	1	0	0	0	3	0	87	0	0	0	0	0	2	0	0	0	0
14'	3	0	0	0	2	5	0	0	9	0	57	0	0	0	0	0	23	0	0	0	0
15	7	2	0	0	0	1	0	0	24	0	23	0	0	0	0	0	38	2	1	0	0
17	4	1	0	0	0	4	0	0	20	0	28	0	0	0	0	0	41	2	0	0	0
18	4	2	1	0	0	4	0	0	27	0	15	0	0	0	0	0	44	0	0	0	0
19	4	1	1	0	2	3	0	0	17	0	17	0	0	0	0	0	54	1	0	0	0
20	5	2	0	0	0	2	0	0	22	0	11	0	0	0	0	0	57	0	0	0	0
22	2	1	0	0	2	4	0	0	20	0	11	0	0	0	0	0	58	2	0	0	0
23	3	1	0	0	1	2	0	0	59	0	8	0	0	0	0	0	16	9	0	0	0
25	3	3	0	0	4	2	0	0	27	0	10	0	0	1	0	0	43	6	1	0	0
26	10	3	1	1	1	16	0	0	3	0	57	0	0	0	0	0	5	3	0	0	0
27	4	1	2	0	7	4	1	0	5	0	29	0	1	1	0	0	43	2	0	0	0
28	2	2	0	0	0	2	0	0	15	0	9	0	0	0	0	0	68	0	0	0	0
30	2	1	0	0	3	1	0	0	34	0	7	0	0	0	0	0	47	3	0	0	0
31	2	1	0	0	2	2	0	0	32	0	0	0	0	0	0	0	57	4	0	0	0
32	1	1	0	0	3	2	0	0	34	0	4	0	0	0	0	0	41	13	0	0	0
36	5	1	1	0	1	3	0	0	19	0	22	0	0	0	0	0	46	0	0	0	0
37	1	3	0	0	1	4	0	0	34	0	5	0	0	0	0	0	49	1	0	0	0
38	1	1	1	0	3	0	0	0	37	0	4	0	0	0	0	0	39	11	1	0	0

Supplementary Table 3 Bulk petrography composition counts for samples in this study. For sample information, please refer to Figure 1 and Supplementary Table 1.

Sample #	Q	F	Lv	Lc	Ls	Lm	Lu	mica	HM
1	63	9	0	11	4	10	0	0	4
2	58	22	0	3	6	10	0	0	1
3	63	14	1	3	1	16	0	1	1
4	54	20	0	3	7	14	0	0	1
5	45	25	2	7	2	14	0	0	5
9	48	23	1	12	1	8	0	2	5
12	72	21	0	3	1	1	0	0	2
14'	52	38	2	2	2	1	0	1	2
15	50	33	0	5	0	2	0	1	8
17	54	27	0	6	1	2	0	1	10
18	49	26	0	9	1	4	0	5	6
19	50	24	2	9	6	4	0	3	1
20	47	28	0	10	0	7	0	2	5
22	49	26	1	11	0	5	0	2	5
23	55	21	1	10	3	6	0	0	3
28	41	20	0	13	0	9	0	9	7
30	48	29	0	13	0	2	0	1	5
31	34	30	7	10	2	7	0	6	4
36	36	26	0	16	5	6	0	4	8
37	49	14	2	19	4	3	0	4	5

Q: quartz; F: feldspar; L: lithic fragments (Lv: volcanic; Lc: carbonate; etamorphic; Lu: ultramafic); HM: heavy mineral

Supplementary References

1 Vermeesch, P. On the visualisation of detrital age distributions. *Chem. Geol.* **312**, 190-194, doi:10.1016/j.chemgeo.2012.04.021 (2012).

2 Vermeesch, P. Multi-sample comparison of detrital age distributions. *Chem. Geol.* **341**, 140-146, doi:http://dx.doi.org/10.1016/j.chemgeo.2013.01.010 (2013).

3 Vermeesch, P. & Garzanti, E. Making geological sense of 'Big Data' in sedimentary provenance analysis. *Chem. Geol.* **409**, 20-27 (2015).

4 Yang, J. *et al.* Episodic crustal growth of North China as revealed by U-Pb age and Hf isotopes of detrital zircons from modern rivers. *Geochim. Cosmochim. Acta* **73**, 2660-2673, doi:http://dx.doi.org/10.1016/j.gca.2009.02.007 (2009).

5 Kong, P., Jia, J. & Zheng, Y. Time constraints for the Yellow River traversing the Sanmen Gorge. *Geochem. Geophys. Geosyst.* **15**, 395-407 (2014).

6 Stevens, T. *et al.* Genetic linkage between the Yellow River, the Mu Us desert, and the Chinese Loess Plateau. *Quat. Sci. Rev.* **78**, 355-368 (2013).

7 Pullen, A. *et al.* Qaidam Basin and northern Tibetan Plateau as dust sources for the Chinese Loess Plateau and paleoclimatic implications. *Geology* **39**, 1031-1034, doi:10.1130/g32296.1 (2011).

8 Nie, J. *et al.* Provenance of the upper Miocene-Pliocene Red Clay deposits of the Chinese loess plateau. *Earth Planet. Sci. Lett.* **407**, 35-47 (2014).
